# Immune-Related Adverse Events Associated With Outcomes in Patients With NSCLC Treated With Anti-PD-1 Inhibitors: A Systematic Review and Meta-Analysis

**DOI:** 10.3389/fonc.2021.708195

**Published:** 2021-09-15

**Authors:** Zhe Zhao, Xinfeng Wang, Jinghan Qu, Wei Zuo, Yan Tang, Huijuan Zhu, Xiaoguang Chen

**Affiliations:** ^1^Department of Pharmacy, Peking Union Medical College Hospital, Chinese Academy of Medical Sciences & Peking Union Medical College, Beijing, China; ^2^State Key Laboratory of Bioactive Substrate and Function of Natural Medicine, Institute of Materia Medica, Chinese Academy of Medical Sciences & Peking Union Medical College, Beijing, China; ^3^Department of Thoracic Surgery, National Cancer Center/Cancer Hospital, Chinese Academy of Medical Sciences and Peking Union Medical College, Beijing, China; ^4^Department of Endocrinology, Key Laboratory of Endocrinology of National Health Commission, Peking Union Medical College Hospital, Chinese Academy of Medical Sciences & Peking Union Medical College, Beijing, China

**Keywords:** immune-related adverse event, non-small cell lung cancer, PD-1 inhibitor, outcome, prognosis

## Abstract

**Background and Objective:**

Although anti-programmed cell death protein 1 (PD-1) antibodies have exerted remarkable anticancer activity in non-small cell lung cancer (NSCLC), it remains a challenge to identify patients who can benefit from these treatments. Immune-related adverse events (irAEs) may be associated with improved clinical outcomes after immune checkpoint inhibition. However, no conclusive evidence of this correlation has been summarized in patients with NSCLC receiving PD-1 inhibitors. We performed a systematic review and meta-analysis to evaluate the association between irAEs induced by anti-PD-1 antibodies and clinical outcomes in patients with NSCLC.

**Methods:**

Various databases were searched from their inception to January 9, 2021, followed by screening of eligible studies. Hazard ratios were used for the pooled analysis of overall survival (OS) and progression-free survival (PFS), while odds ratios (ORs) were utilized to pool objective response rates (ORRs) and disease control rates (DCRs). A random-effects model was applied to all analyses.

**Results:**

A total of 26 cohorts, including 8,452 patients with NSCLC receiving anti-PD-1 antibodies, were enrolled in the study. Significantly improved OS (HR: 0.51; 95% CI: 0.44-0.60; *P* < 0.01) and PFS (HR: 0.50; 95% CI: 0.43-0.58; *P* < 0.01) were found to be correlated with irAEs. In addition, patients with NSCLC who developed irAEs after PD-1 inhibition demonstrated better responses to therapies, confirmed by pooled ORs of ORRs (OR: 3.41; 95% CI: 2.66-4.35; *P* < 0.01) and DCRs (OR: 4.08; 95% CI: 2.30-7.24; *P* < 0.01). Furthermore, subgroup analysis suggested that both skin and endocrine irAEs are closely correlated with a reduced risk of death, whereas pulmonary irAEs showed no association with longer OS.

**Conclusions:**

In patients with NSCLC treated with anti-PD-1 therapies, the presence of irAEs was strongly correlated with better survival and response, suggesting its potential role as a predictive biomarker for outcomes after PD-1 inhibition.

## Introduction

In recent decades, immune checkpoint inhibitors (ICIs) targeting programmed cell death protein 1 (PD-1), programmed death-ligand 1 (PD-L1), and cytotoxic T lymphocyte-associated protein 4 (CTLA-4) have revolutionized the treatment landscape for patients with advanced cancer (1). Anti-PD-1 antibodies (nivolumab and pembrolizumab), which have significant anticancer activity, have garnered approvals from the U.S. Food and Drug Administration for various malignancies, including advanced non-small cell lung cancer (NSCLC), melanoma, head and neck squamous cell carcinoma, renal cell carcinoma, and urothelial carcinoma (2).

Nevertheless, the efficacy of anti-PD-1 drugs varies among individuals, only a fraction of whom benefit from immune checkpoint inhibition. Among all cancer types, previously treated NSCLC exhibited a relatively low response rate to PD-1 inhibitors (<20%) (3–6). Therefore, there is an urgent need to establish predictive biomarkers to identify patients with NSCLC who may benefit from PD-1 inhibition. Several predictive approaches have recently been developed for NSCLC treatment, including biomarkers of PD-L1 expression (6, 7), tumor-infiltrating lymphocytes (8), and tumor mutation burden (9). While these biomarkers were developed primarily to focus on the histological or molecular features of the tumor, evidence for predictive capacity of other clinical characteristics is unclear.

Recent studies have demonstrated some correlations between immune-related adverse events (irAEs) and outcomes after ICI treatments. IrAEs are inflammatory side effects related to the activation of the immune system that are triggered by an immune checkpoint blockade, with most involving the skin, endocrine glands, gastrointestinal tract, liver, and lungs (10). In a recent pooled analysis of 30 studies and 4,324 patients, irAEs were shown to predict favorable responses and survival in patients with solid tumors receiving various ICI treatments (11). In addition, another review of 48 clinical trials of nivolumab, used to treat multiple solid tumors, revealed that the objective response rates (ORRs) of nivolumab were positively associated with incidence rates of gastrointestinal, skin, and endocrine irAEs (12). In a retrospective analysis of 1,010 patients with NSCLC treated with pembrolizumab, irAEs were shown to be significantly related to higher ORRs and better progression-free survival (PFS) and overall survival (OS) (13). However, no existing articles have comprehensively summarized a conclusive association between irAEs and the outcomes of anti-PD-1 regimens in patients with NSCLC. Hence, our current study involved a systematic review and pooled analyses of the literature to reveal possible correlations between the irAEs induced by PD-1 blockade and favorable clinical outcomes in patients with NSCLC.

## Materials and Methods

### Search Strategy

We performed a literature search of the PubMed, EMBASE, and the Cochrane Library databases from their inception to January 9, 2021 for published studies assessing prognostic effects of irAEs in patients with NSCLC receiving anti-PD-1 regimens. The search strategy was developed by combining different descriptions of irAEs, various prognostic outcomes, keywords specific to NSCLC, and currently available anti-PD-1 antibodies. Detailed keywords used for the search are listed in **Supplementary Table S1**. Additionally, we screened studies included in two recent systematic reviews (11, 14) and identified 13 related published articles.

### Study Selection

All the research was independently screened by two investigators to select eligible studies for further analysis. We only included studies that met the following criteria: (1) full text original research including patients diagnosed with NSCLC receiving anti-PD-1 treatment; (2) published articles in the English language; and (3) reported correlations between irAEs and clinical outcomes (OS, PFS, or ORR). We excluded case reports, reviews, meta-analyses, systematic reviews, conference abstracts, and correspondence letters. In addition, studies that included patients with another type of cancer or who were treated with other ICIs were also excluded.

### Data Extraction

The following data were extracted from each study: name of the first author, year of publication, patient number, study type, median time of follow-up, country or area of study, irAE type and grade, irAE evaluation criteria, drugs administered, and any correlations between irAEs and ICI treatment outcomes (survival data or ORRs). The Newcastle-Ottawa Scale (NOS), ranging from 0 to 9, was applied as a quality assessment of all included studies.

### Statistical Analysis

To evaluate the association between irAEs and clinical outcomes, hazard ratios (HRs) with 95% confidence intervals (CIs) were used for survival data (OS or PFS), while odds ratios (ORs) were calculated for ORRs and disease control rates (DCRs). The heterogeneity among the different studies was assessed by the Cochrane’s χ^2^ and Higgins and Thompson’s *I*^2^ statistic (15). For heterogeneity analysis, P value < 0.05 studies were considered as significant heterogeneity. *I*^2^ values < 50%, 50-75%, and > 75% were respectively defined as low, moderate, and high heterogeneity. For pooled analysis, a random-effects model was utilized. Funnel plots were used to assess any publication bias. In this study, *P* values less than 0.05 were considered statistically significant. All analyses were performed using the “meta” package of the R software (V3.6.2).

## Results

### Characteristics of Eligible Studies

**Figure 1:**
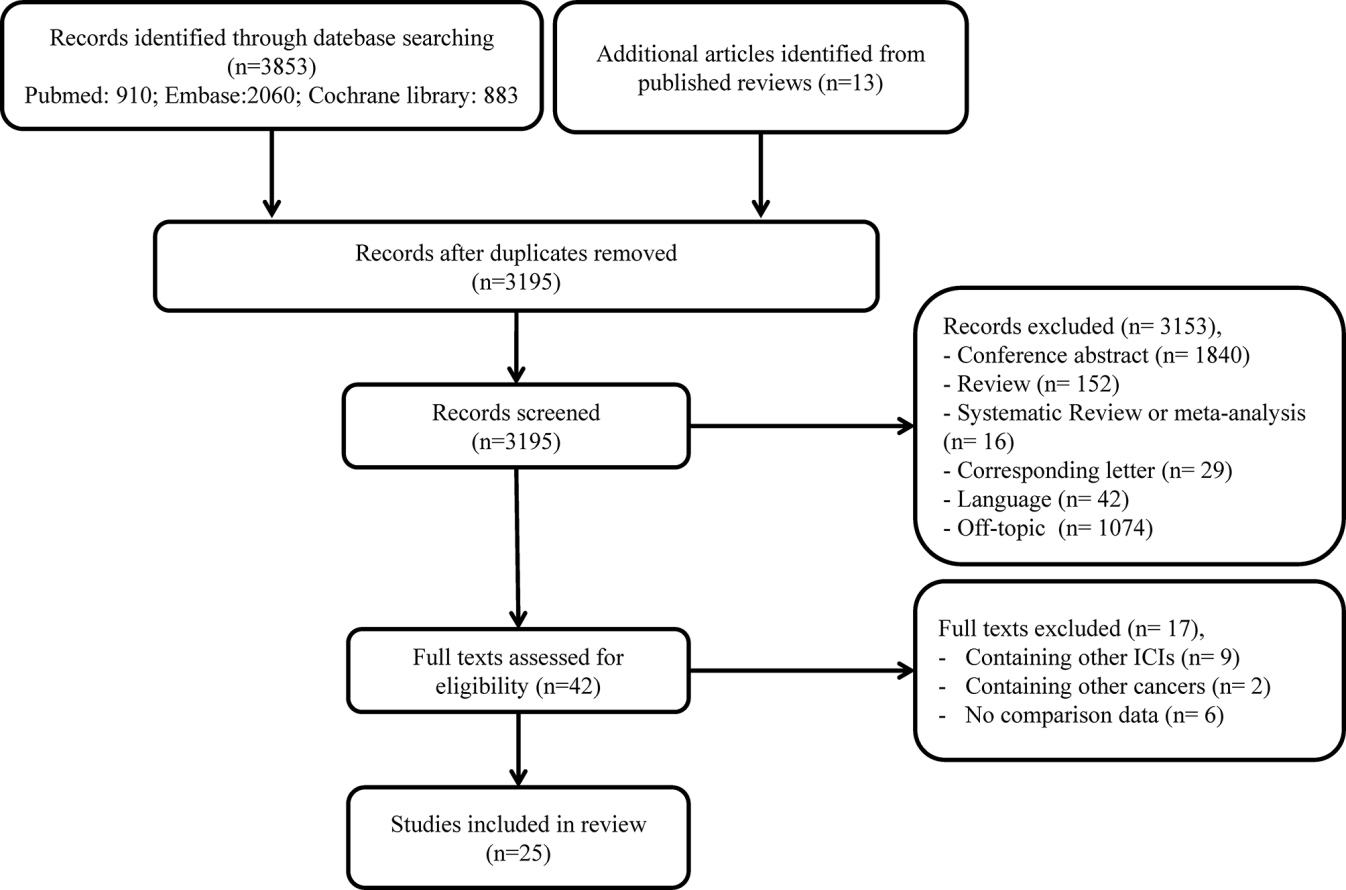
Study selection flow chart.

A total of 3,866 studies were identified in our initial search. After the removal of duplicate records, 3,195 were left for screening. Thereafter, 3,153 articles were excluded due to irrelevant titles or abstracts. The full text of the remaining 42 studies was further assessed for eligibility, and 17 additional publications were excluded. Eventually, 25 articles, including 8,452 patients with confirmed NSCLC receiving anti-PD-1 treatment, were enrolled in our meta-analysis (13, 16–39). The process of study selection is illustrated in **Figure 1**.

The characteristics of these selected articles are listed in **Table 1** and **Supplementary Table S2**. As one article included two independent cohorts, we are presenting them as two separate studies (26). The 26 included studies consisted of 21 retrospective cohorts and 5 prospective cohorts. In 18 studies, clinical outcomes for patients with and without any irAEs were compared. The other eight cohorts included specific adverse events (AEs), including skin reactions (two studies), pneumonitis (three studies), and thyroid dysfunction (three studies). The average incidence of irAEs triggered by PD-1 blockade was 34.9%, which varied from 10% to 67%. In 12 cohorts, patients were treated with nivolumab, while pembrolizumab was administered in six studies. Additionally, eight studies included patients receiving either nivolumab or pembrolizumab monotherapy. Some other detailed clinical features of the enrolled NSCLC patients in each study were illustrated in **Supplementary Table S2**, including clinical stage, histological type, PD-L1 expression status and driver gene mutation information.

**Table 1:** Characteristics of included studies.

Author/year	N	Country	Study type	Follow up (months)	Type of toxicity/criteria	% irAEs	Drug	OS (HR, 95%CI)	PFS (HR, 95%CI)	ORR	Analysis	NOS
Ahn/2019	155	Korea	retrospective	NR	any G1-4/CTCAE v4.0	61.9	P N	0.38 (0.23-0.64)	0.37 (0.23-0.58)	41.2 *vs.* 26.7	UVA	6
Aso/2020	155	Japan	retrospective	NR	skin reaction all grades/CTCAE v4.0	58.1	P N	0.34 (0.20–0.60)	0.38 (0.25-0.58)	57 *vs.* 19	UVA	6
Baldini/2020	1959	Italy	retrospective	NR	any G1-4/CTCAE v4.0	17.8	N	0.60 (0.51-0.71)	0.69 (0.60-0.79)	27.2 *vs.* 16.5	UVA	7
Barlesi/2020	1420	France	prospective cohort	18	any G1-4/-	34.9	N	0.55 (0.48–0.64)	–	–	UVA	8
Barron/2020	101	Mexico	retrospective	9.22	pneumonitis G≥2/CTCAE v4.0	21.8	P N	2.48 (1.18−5.23)	–	–	UVA	8
Cortellini/2019	559	Italy	retrospective	11.2	any G1-4/CTCAE v4.0	41.3	P N	0.47 (0.36-0.60)	0.53 (0.42-0.66)	46.5 *vs.* 25.7	UVA	7
Cortellini/2020	1010	Italy	retrospective	14.8	any G1-4/CTCAE v4.0	32.9	P	0.39 (0.30-0.51)	0.48 (0.39-0.59)	61.5 *vs.* 41.3	UVA	9
Fujimoto/2018	613	Japan	retrospective	NR	pneumonitis G3-5/CTCAE v4.0	10	N	–	0.71 (0.52–0.97)	37 *vs.* 18	MVA	4
Fukihara/2019	170	Japan	retrospective	9.9	pneumonitis G1-5/CTCAE v4.0	16	P N	–	–	30 *vs.* 24		8
Haratani/2018	134	Japan	retrospective	NR	any all grades/-	51	N	0.54 (0.29-0.97)	0.28 (0.10-0.67)	–	MVA	6
Hasan/2016	41	Switzerland	retrospective	NR	skin reaction Grade 1-2/CTCAE v4.0	17	N	–	–	71.4 *vs.* 21.9		4
Hosoya/2020	148	Japan	retrospective	NR	any G1-4/CTCAE v4.0	27	P	–	0.55 (0.31-0.98)	77 *vs.* 44	UVA	6
Hosoya/2020	76	Japan	prospective cohort	NR	any G1-4/CTCAE v4.0	49	N	0.92 (0.47-1.79)	0.60 (0.36-0.99)	39 *vs.* 13	UVA	6
Kim/2018	58	Korea	prospective cohort	3	thyroid disfunction all grades/-	32.7	P N	0.11 (0.01-0.92)	0.38 (0.17-0.85)	31.6 *vs.* 10.3	MVA	7
Ksienski/2019	190	Canada	retrospective	6.1	any G1-2/-	34.7	P	0.66 (0.29-1.48)	–	–	MVA	6
Lim/2020	299	Korea	retrospective	30.1	any G1-4/CTCAE v4.0	32	N	0.44 (0.29-0.67)	0.46 (0.35-0.62)	32 *vs.* 11	UVA	7
Lisberg/2018	97	US	retrospective	NR	any G1-4/CTCAE v4.0	40	P	0.72 (0.49-1.05)	0.62 (0.4-0.96)	38.5 *vs.* 8.9	MVA	6
Naqash/2020	531	US	retrospective	NR	any G1-4/CTCAE v4.0	33	N	0.66 (0.52–0.82)	0.68 (0.55–0.85)	40.1 *vs.* 14.1	UVA	5
Noguchi/2020	94	Japan	retrospective	9.4	any G1-4/CTCAE v4.0	67	P	–	0.24 (0.13-0.42)	–	UVA	6
Osorio/2017	51	US	retrospective	NR	thyroid disfunction all grades/CTCAE v4.0	21	P	0.29 (0.09-0.94)	0.58 (0.27-1.21)	–	UVA	5
Ricciuti/2019	195	Italy	retrospective	26	any G1-4/CTCAE v4.0	43.6	N	0.33 (0.23-0.47)	0.41 (0.30-0.57)	43.5 *vs.* 10	UVA	8
Sato/2018	38	Japan	prospective cohort	5.6	any G1-4/CTCAE v4.0	36.8	N	–	0.10 (0.02-0.37)	63.6 *vs.* 7.4	UVA	6
Suh/2018	54	Korea	retrospective	26.2	any all grades/CTCAE v4.0	22.2	P N	0.48 (0.20-1.14)	0.5 (0.22-1.13)	66.6 *vs.* 23.8	UVA	8
Teraoka/2017	43	Japan	prospective cohort	NR	any G1-4/CTCAE v4.0	44.2	N	–	–	37 *vs.* 17	UVA	5
Toi/2018	70	Japan	retrospective	NR	any G1-4/CTCAE v4.0	40	N	–	0.43 (0.21-0.83)	57 *vs.* 12	UVA	5
Zhou/2021	191	China	retrospective	NR	thyroid disfunction all grades/CTCAE v5.0	20.9	P N	0.33 (0.20–0.57)	–	–	MVA	6

CI, confidence interval; CTCAE, Common Terminology Criteria for Adverse Events; HR, hazard ratio; irAEs, immune-related adverse events; MVA, multivariate analysis; N, nivolumab; NR, not reported; NOS, Newcastle-Ottawa Scale; ORR, objective response rate; OS, overall survival; P, pembrolizumab; PFS, progression-free survival; UVA, univariate analysis.

### Correlation Between irAEs and Survival Results

**Figure 2:**
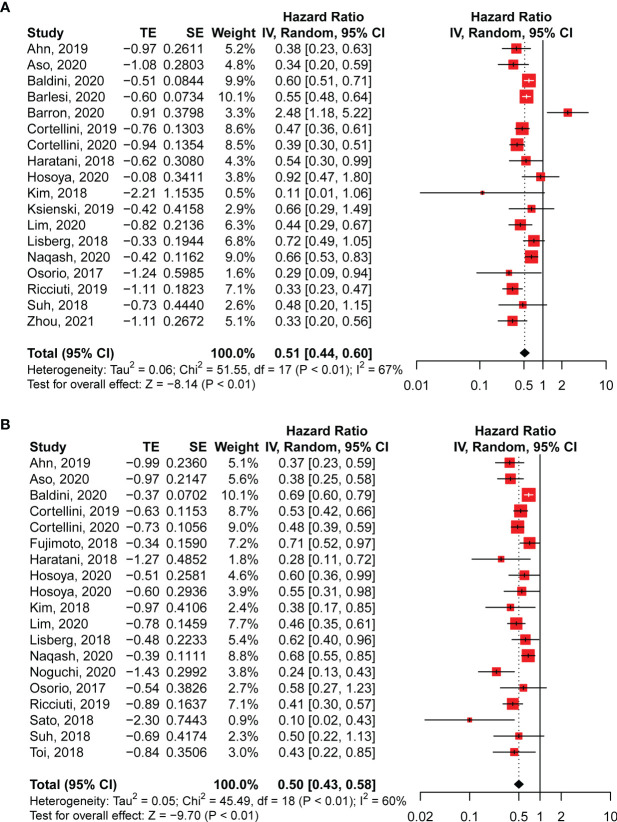
Pooled hazard ratios of overall survival **(A)** and progression-free survival **(B)** in patients with NSCLC with and without irAEs treated with anti-PD-1 antibodies. CI, confidence interval.

The occurrence of irAEs in patients with NSCLC treated with anti-PD-1 antibodies was associated with better survival. The pooled OS data from the 18 studies enrolled in our analysis revealed a significantly lower risk of death in patients with irAEs (HR: 0.51; 95% CI: 0.44–0.60; *P* < 0.01; **Figure 2A**). Meanwhile, moderate but significant heterogeneity was observed in the pooled OS data (*I*^2^ = 67%, *P* < 0.01; **Figure 2A**). Correspondingly, significantly improved PFS correlated with the existence of irAEs (HR: 0.50; 95% CI: 0.43–0.58; *P* < 0.01; **Figure 2B**). For the PFS analysis, pooled HRs also showed moderate heterogeneity (*I*^2^ = 60%, *P* < 0.01; **Figure 2B**).

### Correlation Between irAEs and Responses to PD-1 Blockade

**Figure 3:**
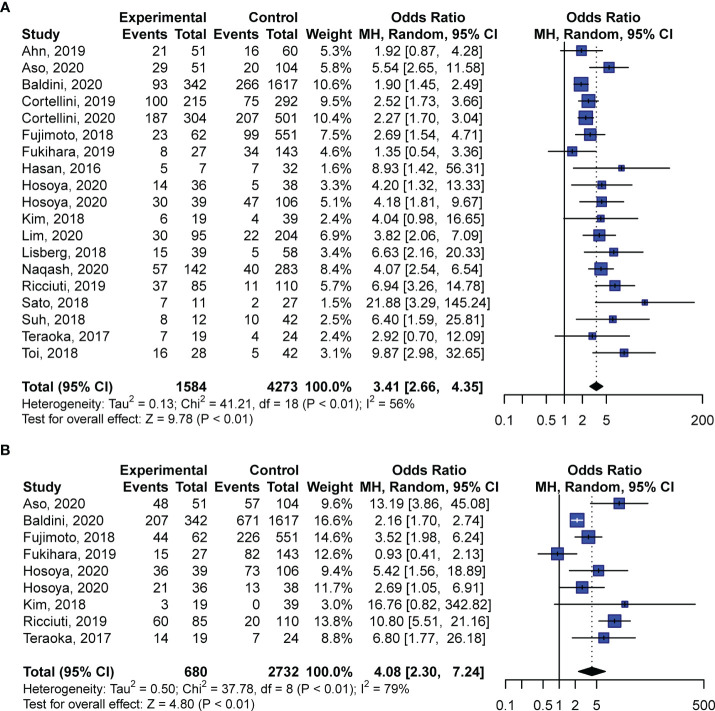
Pooled odds ratios of objective response rates **(A)** and disease control rates **(B)** in patients with NSCLC with and without irAEs treated with anti-PD-1 antibodies. CI, confidence interval.

Further pooled analyses of ORRs and DCRs revealed remarkably higher responses to anti-PD-1 inhibition in patients who exhibited irAEs. Among all the included studies, 19 studies compared ORRs between patients with and without irAEs, whereas only nine cohorts investigated DCRs. For ORR analyses, we found that irAEs were significantly related to higher rates of objective responses to PD-1 blockade (OR: 3.41; 95% CI: 2.66–4.35; *P* < 0.01; **Figure 3A**) with moderate heterogeneity (*I*^2^ = 56%, *P* < 0.01; **Figure 3A**). Likewise, pooled ORs of DCRs demonstrated that patients exhibiting irAEs had better responses to anti-PD-1 regimens than patients without irAEs (OR: 4.08; 95% CI: 2.30–7.24; *P* < 0.01; **Figure 3B**). The analyses of DCRs showed high heterogeneity (*I*^2^ = 79%, *P* < 0.01; **Figure 3B**).

### Publication Bias and Study Quality Assessment

Begg’s funnel plots along with Egger’s tests (*P* = 0.5479) illustrated that the pooled analysis of OS in this study did not have any obvious publication bias (**Supplementary Figure S1**). However, possible publication bias existed in the analyses of PFS (*P* = 0.0041; **Supplementary Figure S2**) and ORR results (*P* = 0.0010; **Supplementary Figure S3**). The number of studies with DCR results did not meet the level of publication bias. In the enrolled 26 studies, the median NOS score was 6 (range: 4–9). Over one-half of the studies (14/26) did not report the follow-up time for the cohorts, lowering their NOS scores. In addition, we performed sensitivity analysis by omitting one study at a time for the pooled analyses to evaluate the potential influence of each study on our conclusions. The results showed that not a single study affected the association between better outcome and irAEs (**Supplementary Figure S4**).

### Subgroup Analysis

**Figure 4:**
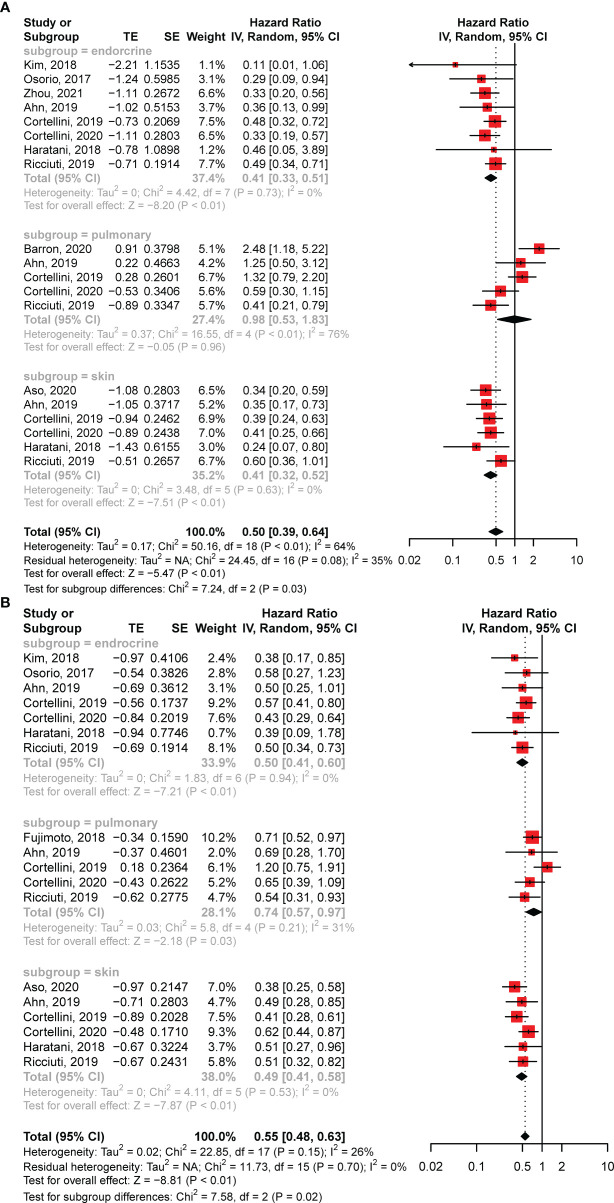
Forest plots of subgroup analysis. **(A)** The association between overall survival and different toxicity types in patients with NSCLC treated with anti-PD-1 antibodies. **(B)** The association between progression-free survival and various irAEs in patients with NSCLC receiving anti-PD-1 antibodies. CI, confidence interval.

To further investigate the influence of different AEs, we performed subgroup analyses for pulmonary, skin, and endocrine irAEs. In addition to the aforementioned eight studies of specific irAEs (17, 20, 22, 23, 25, 27, 33, 39), we also extracted survival data from the other five articles that reported HRs for these three AEs (13, 16, 21, 24, 34). The analysis revealed that skin (HR: 0.41; 95% CI: 0.32–0.52; *P* < 0.01) and endocrine (HR: 0.41; 95% CI: 0.33–0.51; *P* < 0.01) irAEs were significantly associated with longer OS, whereas pulmonary irAEs showed no correlation (HR: 0.98; 95% CI: 0.53–1.83; *P* = 0.96) (**Figure 4A**). In addition, the subgroup analysis of PFS found that all three irAEs had significant associations with better disease control (**Figure 4B**).

More subgroup analyses based on the features of the included studies were also performed. The pooled analyses for prospective studies suggested that irAEs were associated with better PFS (HR: 0.36; 95% CI: 0.16–0.81; *P* = 0.01) but not OS (HR: 0.60; 95% CI: 0.35–1.03; *P* = 0.07) (**Supplementary Figure S5**). For retrospective studies, the occurrence of irAEs was found to have correlations with better OS (HR: 0.50; 95% CI: 0.42–0.61; *P* < 0.01) and PFS (HR: 0.51; 95% CI: 0.44–0.59; *P* < 0.01) (**Supplementary Figure S5**). Also, the subgroup analyses for Asian and non-Asian studies both showed that the presence of irAEs was correlated with longer OS and PFS (**Supplementary Figure S6**). Additionally, the subgroup analyses separated by the anti-PD-1 drugs used in the studies revealed that the association between irAEs and better outcomes existed no matter nivolumab or pembrolizumab was used for treatment (**Supplementary Figure S7**).

## Discussion

This is the first and most comprehensive review of studies investigating the association between irAEs and clinical outcomes of patients with NSCLC receiving anti-PD-1 antibodies. In our pooled analysis of the 26 cohorts, we report a strong correlation between the presence of irAEs and improved patient response and prognosis, suggesting the significance of irAEs as a predictor of anti-PD-1 therapeutic efficacy in patients with NSCLC.

**Figure 5:**
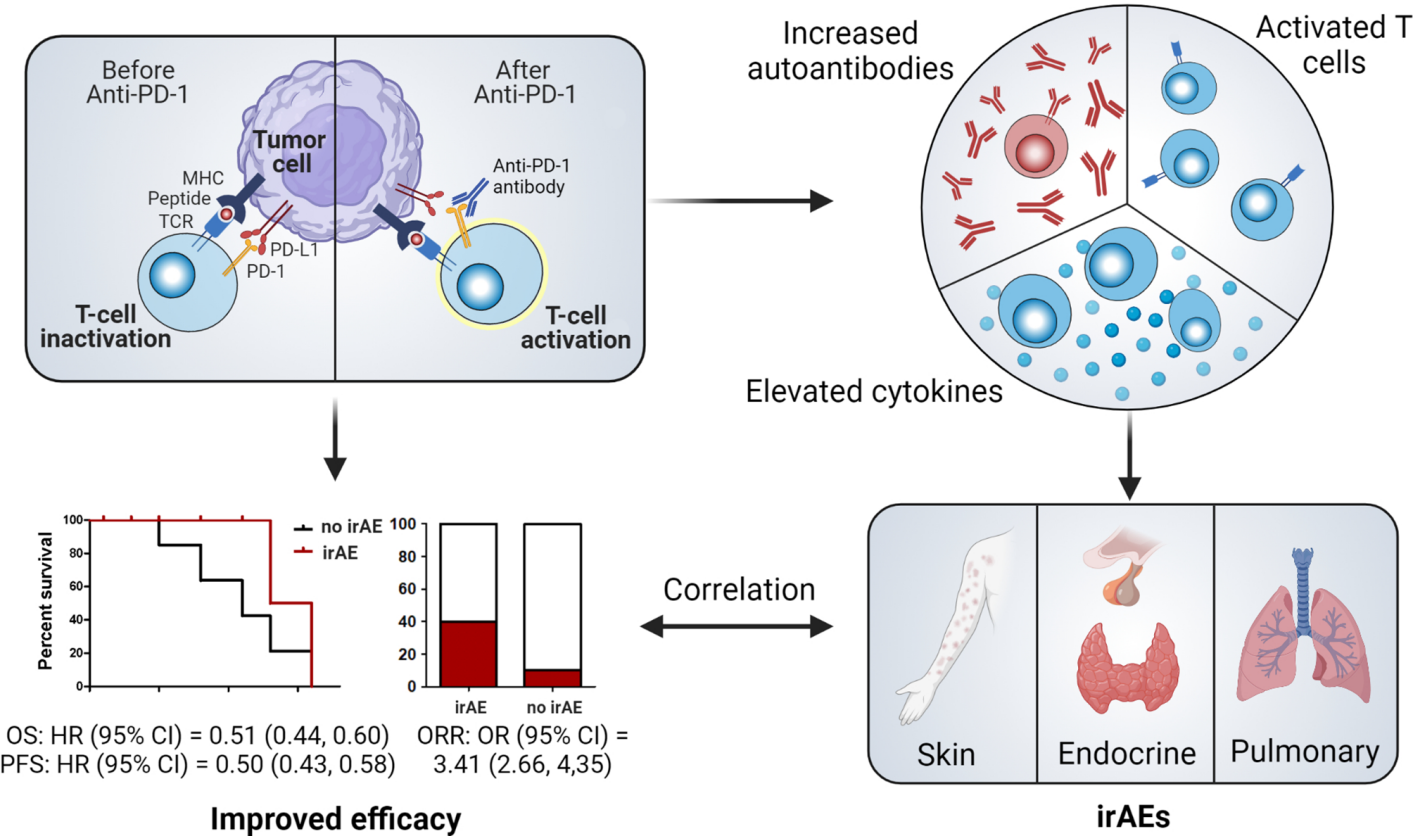
Illustration of potential mechanisms of irAE occurrence and their relationship with the efficacy of immune checkpoint blockade. CI, confidence interval; MHC, major histocompatibility complex; HR, hazard ratio; irAE, immune-related adverse event; OR, odds ratio; ORR, objective response rate; PD-1, programmed cell death protein 1; PFS, progression-free survival; TCR, T-cell receptor.

In addition to the recognition of antigens combined with major histocompatibility complexes by T-cell receptors, the stimulation of B7-CD28, known as the costimulatory signal, is indispensable for T-cell activation (40). To avoid the overactivation of T-cells and restrict their autoimmune responses, CTLA-4 (on T-cells) (41, 42) and PD-1 (on T-cells, B-cells, monocytes, natural killer cells, and dendritic cells) exert inhibitory effects by binding to their ligands (PD-L1 or PD-L2) (43). However, in tumor tissues, these immune checkpoint pathways help cancer cells escape the immune system (44). Therefore, ICIs are used to block the overactivation of these pathways to enhance the antitumor immune responses mediated by T-cells (**Figure 5**). Two anti-PD-1 inhibitors (nivolumab and pembrolizumab), which exhibit outstanding efficacy to prolong cancer patient survival, have been approved for the treatment of NSCLC.

Apart from their anticancer efficacy, ICIs also trigger autoimmunity, which results in irAEs (45). Although the precise pathophysiology of irAE onset is still unclear, the possible mechanisms may involve the overactivation of T-cells, stimulation of autoantibodies, and elevation of cytokine levels (**Figure 5**) (10). Therefore, the occurrence of irAEs demonstrates that a patient’s immune responses have been activated and that irAE development might be an effective biomarker of ICI efficacy. However, whether this clinical event can help predict responses to ICIs requires additional evidence. Certain irAEs specific to some cancer types have been found to be more strongly associated with improved clinical outcomes. For example, vitiligo, an irAE that mainly occurs in melanoma patients treated with ICIs but rarely in patients with other cancers, has been shown to be closely correlated with favorable outcomes (46, 47). Except for this well-established correlation, other real-world studies have failed to provide definitive associations (37, 48–50). Recent systematic reviews and meta-analyses have suggested the presence of significant associations between irAEs and beneficial clinical outcomes in a pan-cancer setting (11, 51). However, these studies involve patients with different cancers receiving various ICIs, which contradicts the principles of personalized medicine. Further comprehensive research of patients with specific cancer types receiving specific ICIs is thus urgently needed for clinical application.

To avoid such heterogeneity and improve study comparability, we focused our analysis on patients with NSCLC treated with anti-PD-1 antibodies. Consistent with the subgroup analysis results of NSCLC from other systematic reviews (11, 51, 52), our research revealed that the occurrences of irAEs in patients with NSCLC treated with anti-PD-1 antibodies were closely associated with improved clinical outcomes, including OS, PFS, ORRs, and DCRs. Our results demonstrated that patients with NSCLC who developed any irAE after anti-PD-1 treatment showed a 50% reduction in the risks of death and disease progression compared to those without any AEs related to ICIs. Additionally, patients with irAEs exhibited better responses to immune checkpoint blockade. These data indicate that irAEs play a critical role in predicting the efficacy of PD-1 therapies in patients with NSCLC. Since these findings are concluded in a specific cancer, our current investigation is closer to clinical usage than existing studies.

We also analyzed the correlations of pulmonary, skin, and endocrine irAEs with survival data. Strikingly, skin and endocrine AEs predicted better survival, whereas pulmonary irAEs were only associated with prolonged PFS but not with OS. Another meta-analysis enrolling patients with various types of cancers showed that various AEs (except pneumonitis) were correlated with improved clinical outcomes (11). In addition, a recent systematic review calculated the correlations between ORRs after nivolumab treatment and incidences of different nivolumab-related irAEs in patients with different solid tumors, revealing that the ORRs were positively associated with skin (r = 0.79, *P* < 0.001) and endocrine (r = 0.44, *P* = 0.05) irAEs but not with pulmonary irAEs (12). These results confirm our findings from the subgroup analysis. Although antitumor immune responses in patients with lung cancer and pulmonary irAEs are similar, suggesting that pneumonitis may be a favorable biomarker for the efficacy of ICIs in NSCLC, the predictive effects of these AEs may be compromised by several reasons. First, the incidence rates of pulmonary irAEs are low in patients receiving PD-1 antibodies (53, 54) or other ICIs (55), which would cause a disparity between patients with and without immune-related pneumonitis, making it difficult to compare the two groups. Second, pulmonary irAEs are always associated with severe disease and mortality during treatment with ICIs (56), which might also be associated with poor outcomes after immune checkpoint blockade. Taken together, our analysis indicates that endocrine and skin irAEs might be effective predictors of improved outcomes after anti-PD-1 therapies in patients with NSCLC. However, more investigations are needed to determine the specific role of pulmonary irAEs in patients with NSCLC receiving ICIs.

The average incidence of an irAE in our analysis (excluding studies only reporting specific AEs) was 39.4% (ranging from 17.8% to 67.0%) for patients with NSCLC treated with PD-1 inhibitors, consistent with findings from other studies (11). Moreover, our study included both prospective and retrospective cohorts, which better approximate real-world data. All studies were carried out in North America, Asia, and Europe. Although more than half of these enrolled studies were conducted in Asia (15/26), the total number of patients in Asia was only 2,298, which is less than the number of patients in the European studies (5,184 patients). These results indicate that our analysis can be applied to patients with NSCLC receiving anti-PD-1 therapies worldwide. Furthermore, we performed some subgroup analyses based on the characteristics of the eligible studies to assess the impact of these features on the analysis. The results of subgroup analyses were consistent with the findings of all-inclusive meta-analyses, proving that the correlation is robust despite of the heterogeneity between the enrolled studies.

By identifying the correlations between irAEs and better immune responses to anti-PD-1 antibodies, our study emphasizes the significance of monitoring, detecting, and managing irAEs during the course of anti-PD-1 treatments. Patients with NSCLC with few or moderate AEs after treatment with anti-PD-1 antibodies may experience better outcomes than patients without any irAEs. However, the presence of severe irAEs might be unfavorable for patient survival, as these AEs are sometimes life-threatening and affected patients may need to discontinue their ICI therapy. Therefore, close monitoring and early detection of irAEs can help physicians accurately recognize less severe side effects, stratify patients with effective immune responses to PD-1 inhibitors, and prevent irAEs from progressing into more severe AEs. As described in the included studies, patients with common skin irAEs may develop some symptoms like immune-related pruritus, rash, and erythema (24, 34), which can be easy to identify. Some endocrine irAEs following anti-PD-1 therapies include hyper/hypothyroidism with two or more abnormal thyroid function tests (free thyroxine, free triiodothyronine, and thyroid stimulating hormone) (39), and adrenal insufficiency diagnosed by an adrenocorticotropic hormone stimulation test (57). Once irAEs are identified in a patient, appropriate and prompt management can be carried out in a timely manner to improve patient outcomes. Recently, guidelines for the management of irAEs were published (58, 59). Our study highlights the complex but crucial role of irAEs in the use of anti-PD-1 therapy in patients with NSCLC, which may contribute to the update of guideline for NSCLC.

To the best of our knowledge, this study is the first and most comprehensive systematic review and meta-analysis which summarizes and evaluates the correlation between irAE occurrence and clinical outcomes after receiving anti-PD-1 antibodies in NSCLC. Although some other systematic reviews have suggested the association between irAEs and improved clinical response of ICIs, they did not focus on a specific cancer type or a specific kind of ICIs. Therefore, they only summarized partial reports. Fausto et al. (11) included 10 studies regarding NSCLC patients receiving anti-PD-1 treatments in an overall systematic review of solid tumors. Besides, Park et al. (52) concluded the predictive effects of anti-PD-1/L1-associated irAEs for favorable clinical outcomes in a recent systematic review, which only covered 11 studies of NSCLC treated with anti-PD-1 regimens. Recently, Wang et al. (60) reported that irAEs in lung cancer might predict better ICI efficacy, in which 17 lung cancer cohorts treated with anti-PD-1 regimens were included. Compared to these published reviews, we added approximately 9 more cohorts for meta-analysis, making our review more comprehensive and persuasive. Since the effects of different ICIs in various cancers have totally different mechanisms and manifestations, those results concluded from other cancer categories or drugs can hardly be applicable for the cases discussed in our current study. Hence, our results are more important for personalized treatment for NSCLC patients who undergo anti-PD-1 therapies. However, our study still has some limitations. First, publication bias and heterogeneity existed in our analysis, which may be caused by the differences in the characteristics of the included studies. Nevertheless, our subgroup analyses based on these characteristics and sensitivity analysis results suggest that heterogeneity between the included studies have little influence on our main conclusions. Second, most of the studies were retrospective cohort studies because of the scarce number of available prospective studies. Even so, the subgroup analyses for prospective studies suggest a significant correlation between irAE occurrence and better survival. Hence, we hope that our study encourages more prospective investigations of the relationship between irAE occurrence and ICI efficacy. Third, based on the available studies, our analysis demonstrates correlations rather than causal results. Other predictive biomarkers developed on the basis of tumor histological or genomic features may not affect our analysis and results. Nevertheless, the underlying mechanisms of how irAEs can predict outcomes after ICIs and whether other biomarkers have relationships with irAE occurrence require more investigation.

## Conclusions

This study is the first meta-analysis to assess the predictive effects of irAE onset on clinical outcomes for patients with NSCLC receiving anti-PD-1 regimens. We demonstrate a significant correlation between the presence of irAEs and positive prognosis for patients with NSCLC after treatment with anti-PD-1 antibodies, suggesting that irAEs may be a clinical predictive biomarker for efficacy of anti-PD-1 therapy in NSCLC patients.

## Data Availability Statement

The original contributions presented in the study are included in the article/**Supplementary Material**. Further inquiries can be directed to the corresponding authors.

## Author Contributions

Conceptualization, TY, ZH and CX. Writing—original draft preparation, ZZ and WX. Collection and curation of data, ZW and QJ. Revision of the manuscript, TY, ZW, QJ and CX. All authors contributed to the article and approved the submitted version.

## Funding

This work was supported by CAMS Innovation Fund for Medical Sciences (2017-I2M-1-010), The Drug Innovation Major Project (No. 2018ZX09711001-003, China), and CAMS & PUMC Innovation Fund for Graduate (No.2019-1007-23, China).

## Conflict of Interest

The authors declare that the research was conducted in the absence of any commercial or financial relationships that could be construed as a potential conflict of interest.

## Publisher’s Note

All claims expressed in this article are solely those of the authors and do not necessarily represent those of their affiliated organizations, or those of the publisher, the editors and the reviewers. Any product that may be evaluated in this article, or claim that may be made by its manufacturer, is not guaranteed or endorsed by the publisher.

## References

[1] Wilky BA. Immune Checkpoint Inhibitors: The Linchpins of Modern Immunotherapy. Immunol Rev (2019) 290:6–23. doi: 10.1111/imr.12766 31355494

[2] Wu X Gu Z Chen Y Chen B Chen W Weng L. Application of PD-1 Blockade in Cancer Immunotherapy. Comput Struct Biotechnol J (2019) 17:661–74. doi: 10.1016/j.csbj.2019.03.006 PMC6558092 31205619

[3] Gettinger SN Horn L Gandhi L Spigel DR Antonia SJ Rizvi NA. Overall Survival and Long-Term Safety of Nivolumab (Anti-Programmed Death 1 Antibody, BMS-936558, ONO-4538) in Patients With Previously Treated Advanced Non-Small-Cell Lung Cancer. J Clin Oncol (2015) 33:2004–12. doi: 10.1200/jco.2014.58.3708 PMC4672027 25897158

[4] Herbst RS Baas P Kim DW Felip E Pérez-Gracia JL Han JY. Pembrolizumab *Versus* Docetaxel for Previously Treated, PD-L1-Positive, Advanced Non-Small-Cell Lung Cancer (KEYNOTE-010): A Randomised Controlled Trial. Lancet (2016) 387:1540–50. doi: 10.1016/s0140-6736(15)01281-7 26712084

[5] Rizvi NA Mazières J Planchard D Stinchcombe TE Dy GK Antonia SJ. Activity and Safety of Nivolumab, an Anti-PD-1 Immune Checkpoint Inhibitor, for Patients With Advanced, Refractory Squamous Non-Small-Cell Lung Cancer (CheckMate 063): A Phase 2, Single-Arm Trial. Lancet Oncol (2015) 16:257–65. doi: 10.1016/s1470-2045(15)70054-9 PMC5726228 25704439

[6] Garon EB Rizvi NA Hui R Leighl N Balmanoukian AS Eder JP. Pembrolizumab for the Treatment of Non-Small-Cell Lung Cancer. N Engl J Med (2015) 372:2018–28. doi: 10.1056/NEJMoa1501824 25891174

[7] Borghaei H Paz-Ares L Horn L Spigel DR Steins M Ready NE. Nivolumab *Versus* Docetaxel in Advanced Nonsquamous Non-Small-Cell Lung Cancer. N Engl J Med (2015) 373:1627–39. doi: 10.1056/NEJMoa1507643 PMC5705936 26412456

[8] Yu Y Zeng D Ou Q Liu S Li A Chen Y. Association of Survival and Immune-Related Biomarkers With Immunotherapy in Patients With Non-Small Cell Lung Cancer: A Meta-Analysis and Individual Patient-1Level Analysis. JAMA Netw Open (2019) 2:e196879. doi: 10.1001/jamanetworkopen.2019.6879 31290993 PMC6625073

[9] Hellmann MD Ciuleanu TE Pluzanski A Lee JS Otterson GA Audigier-Valette C. Nivolumab Plus Ipilimumab in Lung Cancer With a High Tumor Mutational Burden. N Engl J Med (2018) 378:2093–104. doi: 10.1056/NEJMoa1801946 PMC7193684 29658845

[10] Postow MA Sidlow R Hellmann MD. Immune-Related Adverse Events Associated With Immune Checkpoint Blockade. N Engl J Med (2018) 378:158–68. doi: 10.1056/NEJMra1703481 29320654

[11] Petrelli F Grizzi G Ghidini M Ghidini A Ratti M Panni S. Immune-Related Adverse Events and Survival in Solid Tumors Treated With Immune Checkpoint Inhibitors: A Systematic Review and Meta-Analysis. J Immunother (2020) 43:1–7. doi: 10.1097/cji.0000000000000300 31574022

[12] Xing P Zhang F Wang G Xu Y Li C Wang S. Incidence Rates of Immune-Related Adverse Events and Their Correlation With Response in Advanced Solid Tumours Treated With NIVO or NIVO+IPI: A Systematic Review and Meta-Analysis. J Immunother Cancer (2019) 7:341. doi: 10.1186/s40425-019-0779-6 31801636 PMC6894272

[13] Cortellini A Friedlaender A Banna GL Porzio G Bersanelli M Cappuzzo F. Immune-Related Adverse Events of Pembrolizumab in a Large Real-World Cohort of Patients With NSCLC With a PD-L1 Expression ≥ 50% and Their Relationship With Clinical Outcomes. Clin Lung Cancer (2020) 21:498–508.e2. doi: 10.1016/j.cllc.2020.06.010 32680806

[14] Cortellini A Buti S Agostinelli V Bersanelli M. A Systematic Review on the Emerging Association Between the Occurrence of Immune-Related Adverse Events and Clinical Outcomes With Checkpoint Inhibitors in Advanced Cancer Patients. Semin Oncol (2019) 46:362–71. doi: 10.1053/j.seminoncol.2019.10.003 31727344

[15] Higgins JP Thompson SG. Quantifying Heterogeneity in a Meta-Analysis. Stat Med (2002) 21:1539–58. doi: 10.1002/sim.1186 12111919

[16] Ahn BC Pyo KH Xin CF Jung D Shim HS Lee CY. Comprehensive Analysis of the Characteristics and Treatment Outcomes of Patients With Non-Small Cell Lung Cancer Treated With Anti-PD-1 Therapy in Real-World Practice. J Cancer Res Clin Oncol (2019) 145:1613–23. doi: 10.1007/s00432-019-02899-y PMC6527531 30911841

[17] Aso M Toi Y Sugisaka J Aiba T Kawana S Saito R. Association Between Skin Reaction and Clinical Benefit in Patients Treated With Anti-Programmed Cell Death 1 Monotherapy for Advanced Non-Small Cell Lung Cancer. Oncologist (2020) 25:e536–e44. doi: 10.1634/theoncologist.2019-0550 PMC7066688 32162801

[18] Baldini E Lunghi A Cortesi E Turci D Signorelli D Stati V. Immune-Related Adverse Events Correlate With Clinical Outcomes in NSCLC Patients Treated With Nivolumab: The Italian NSCLC Expanded Access Program. Lung Cancer (2020) 140:59–64. doi: 10.1016/j.lungcan.2019.12.014 31881412

[19] Barlesi F Dixmier A Debieuvre D Raspaud C Auliac JB Benoit N. Effectiveness and Safety of Nivolumab in the Treatment of Lung Cancer Patients in France: Preliminary Results From the Real-World EVIDENS Study. OncoImmunology (2020) 9:1744898. doi: 10.1080/2162402X.2020.1744898 33457089 PMC7790497

[20] Barrón F Sánchez R Arroyo-Hernández M Blanco C Zatarain-Barrón ZL Catalán R. Risk of Developing Checkpoint Immune Pneumonitis and Its Effect on Overall Survival in Non-Small Cell Lung Cancer Patients Previously Treated With Radiotherapy. Front Oncol (2020) 10:570233. doi: 10.3389/fonc.2020.570233 33117699 PMC7550759

[21] Cortellini A Chiari R Ricciuti B Metro G Perrone F Tiseo M. Correlations Between the Immune-Related Adverse Events Spectrum and Efficacy of Anti-PD1 Immunotherapy in NSCLC Patients. Clin Lung Cancer (2019) 20:237–47.e1. doi: 10.1016/j.cllc.2019.02.006 30885550

[22] Fujimoto D Yoshioka H Kataoka Y Morimoto T Kim YH Tomii K. Efficacy and Safety of Nivolumab in Previously Treated Patients With Non-Small Cell Lung Cancer: A Multicenter Retrospective Cohort Study. Lung Cancer (2018) 119:14–20. doi: 10.1016/j.lungcan.2018.02.017 29656747

[23] Fukihara J Sakamoto K Koyama J Ito T Iwano S Morise M. Prognostic Impact and Risk Factors of Immune-Related Pneumonitis in Patients With Non-Small-Cell Lung Cancer Who Received Programmed Death 1 Inhibitors. Clin Lung Cancer (2019) 20:442–50.e4. doi: 10.1016/j.cllc.2019.07.006 31446020

[24] Haratani K Hayashi H Chiba Y Kudo K Yonesaka K Kato R. Association of Immune-Related Adverse Events With Nivolumab Efficacy in Non-Small-Cell Lung Cancer. JAMA Oncol (2018) 4:374–8. doi: 10.1001/jamaoncol.2017.2925 PMC6583041 28975219

[25] Hasan Ali O Diem S Markert E Jochum W Kerl K French LE. Characterization of Nivolumab-Associated Skin Reactions in Patients With Metastatic Non-Small Cell Lung Cancer. Oncoimmunology (2016) 5:e1231292. doi: 10.1080/2162402x.2016.1231292 27999741 PMC5139632

[26] Hosoya K Fujimoto D Morimoto T Kumagai T Tamiya A Taniguchi Y. Association Between Early Immune-Related Adverse Events and Clinical Outcomes in Patients With Non-Small Cell Lung Cancer Treated With Immune Checkpoint Inhibitors. Clin Lung Cancer (2020) 21:e315–e28. doi: 10.1016/j.cllc.2020.01.003 32113737

[27] Kim HI Kim M Lee SH Park SY Kim YN Kim H. Development of Thyroid Dysfunction is Associated With Clinical Response to PD-1 Blockade Treatment in Patients With Advanced Non-Small Cell Lung Cancer. OncoImmunology (2018) 7:e1375642. doi: 10.1080/2162402X.2017.1375642 PMC5739550 29296533

[28] Ksienski D Wai ES Croteau N Freeman AT Chan A Fiorino L. Pembrolizumab for Advanced Nonsmall Cell Lung Cancer: Efficacy and Safety in Everyday Clinical Practice. Lung Cancer (2019) 133:110–6. doi: 10.1016/j.lungcan.2019.05.005 31200816

[29] Lim SM Kim SW Cho BC Kang JH Ahn MJ Kim DW. Real-World Experience of Nivolumab in Non-Small Cell Lung Cancer in Korea. Cancer Res Treat (2020) 52:1112–9. doi: 10.4143/crt.2020.245 PMC7577826 32599984

[30] Lisberg A Tucker DA Goldman JW Wolf B Carroll J Hardy A. Treatment-Related Adverse Events Predict Improved Clinical Outcome in NSCLC Patients on KEYNOTE-001 at a Single Center. Cancer Immunol Res (2018) 6:288–94. doi: 10.1158/2326-6066.Cir-17-0063 PMC6066474 29382669

[31] Naqash AR Ricciuti B Owen DH Florou V Toi Y Cherry C. Outcomes Associated With Immune-Related Adverse Events in Metastatic Non-Small Cell Lung Cancer Treated With Nivolumab: A Pooled Exploratory Analysis From a Global Cohort. Cancer Immunol Immunother (2020) 69:1177–87. doi: 10.1007/s00262-020-02536-5 PMC11027623 32140762

[32] Noguchi S Suminaga K Kaki T Kawachi H Fukao A Terashita S. Correlation of Immune-Related Adverse Events and Effects of Pembrolizumab Monotherapy in Patients With Non-Small Cell Lung Cancer. Lung Cancer (Auckl) (2020) 11:53–7. doi: 10.2147/lctt.S254146 PMC7367935 32765147

[33] Osorio JC Ni A Chaft JE Pollina R Kasler MK Stephens D. Antibody-Mediated Thyroid Dysfunction During T-Cell Checkpoint Blockade in Patients With Non-Small-Cell Lung Cancer. Ann Oncol (2017) 28:583–9. doi: 10.1093/annonc/mdw640 PMC5834017 27998967

[34] Ricciuti B Genova C De Giglio A Bassanelli M Dal Bello MG Metro G. Impact of Immune-Related Adverse Events on Survival in Patients With Advanced Non-Small Cell Lung Cancer Treated With Nivolumab: Long-Term Outcomes From a Multi-Institutional Analysis. J Cancer Res Clin Oncol (2019) 145:479–85. doi: 10.1007/s00432-018-2805-3 PMC11810236 30506406

[35] Sato K Akamatsu H Murakami E Sasaki S Kanai K Hayata A. Correlation Between Immune-Related Adverse Events and Efficacy in Non-Small Cell Lung Cancer Treated With Nivolumab. Lung Cancer (2018) 115:71–4. doi: 10.1016/j.lungcan.2017.11.019 29290265

[36] Suh KJ Kim SH Kim YJ Kim M Keam B Kim TM. Post-Treatment Neutrophil-to-Lymphocyte Ratio at Week 6 is Prognostic in Patients With Advanced Non-Small Cell Lung Cancers Treated With Anti-PD-1 Antibody. Cancer Immunol Immunother (2018) 67:459–70. doi: 10.1007/s00262-017-2092-x PMC11028357 29204702

[37] Teraoka S Fujimoto D Morimoto T Kawachi H Ito M Sato Y. Early Immune-Related Adverse Events and Association With Outcome in Advanced Non-Small Cell Lung Cancer Patients Treated With Nivolumab: A Prospective Cohort Study. J Thorac Oncol (2017) 12:1798–805. doi: 10.1016/j.jtho.2017.08.022 28939128

[38] Toi Y Sugawara S Kawashima Y Aiba T Kawana S Saito R. Association of Immune-Related Adverse Events With Clinical Benefit in Patients With Advanced Non-Small-Cell Lung Cancer Treated With Nivolumab. Oncologist (2018) 23:1358–65. doi: 10.1634/theoncologist.2017-0384 PMC6291330 29934411

[39] Zhou Y Xia R Xiao H Pu D Long Y Ding Z. Thyroid Function Abnormality Induced by PD-1 Inhibitors Have a Positive Impact on Survival in Patients With Non-Small Cell Lung Cancer. Int Immunopharmacol (2021) 91:107296. doi: 10.1016/j.intimp.2020.107296 33360368

[40] Mueller DL Jenkins MK Schwartz RH. Clonal Expansion *Versus* Functional Clonal Inactivation: A Costimulatory Signalling Pathway Determines the Outcome of T Cell Antigen Receptor Occupancy. Annu Rev Immunol (1989) 7:445–80. doi: 10.1146/annurev.iy.07.040189.002305 2653373

[41] Jain N Nguyen H Chambers C Kang J. Dual Function of CTLA-4 in Regulatory T Cells and Conventional T Cells to Prevent Multiorgan Autoimmunity. Proc Natl Acad Sci USA (2010) 107:1524–8. doi: 10.1073/pnas.0910341107 PMC2824392 20080649

[42] Pedicord VA Montalvo W Leiner IM Allison JP. Single Dose of Anti-CTLA-4 Enhances CD8+ T-Cell Memory Formation, Function, and Maintenance. Proc Natl Acad Sci USA (2011) 108:266–71. doi: 10.1073/pnas.1016791108 PMC3017182 21173239

[43] Ishida Y Agata Y Shibahara K Honjo T. Induced Expression of PD-1, a Novel Member of the Immunoglobulin Gene Superfamily, Upon Programmed Cell Death. EMBO J (1992) 11:3887–95. doi: 10.1002/j.1460-2075.1992.tb05481.x PMC556898 1396582

[44] Pauken KE Wherry EJ. Overcoming T Cell Exhaustion in Infection and Cancer. Trends Immunol (2015) 36:265–76. doi: 10.1016/j.it.2015.02.008 PMC4393798 25797516

[45] Weber JS Yang JC Atkins MB Disis ML. Toxicities of Immunotherapy for the Practitioner. J Clin Oncol Off J Am Soc Clin Oncol (2015) 33:2092–9. doi: 10.1200/JCO.2014.60.0379 PMC4881375 25918278

[46] Hua C Boussemart L Mateus C Routier E Boutros C Cazenave H. Association of Vitiligo With Tumor Response in Patients With Metastatic Melanoma Treated With Pembrolizumab. JAMA Dermatol (2016) 152:45–51. doi: 10.1001/jamadermatol.2015.2707 26501224

[47] Teulings HE Limpens J Jansen SN Zwinderman AH Reitsma JB Spuls PI. Vitiligo-Like Depigmentation in Patients With Stage III-IV Melanoma Receiving Immunotherapy and Its Association With Survival: A Systematic Review and Meta-Analysis. J Clin Oncol (2015) 33:773–81. doi: 10.1200/jco.2014.57.4756 25605840

[48] Attia P Phan GQ Maker AV Robinson MR Quezado MM Yang JC. Autoimmunity Correlates With Tumor Regression in Patients With Metastatic Melanoma Treated With Anti-Cytotoxic T-Lymphocyte Antigen-4. J Clin Oncol (2005) 23:6043–53. doi: 10.1200/jco.2005.06.205 PMC1473965 16087944

[49] Horvat TZ Adel NG Dang TO Momtaz P Postow MA Callahan MK. Immune-Related Adverse Events, Need for Systemic Immunosuppression, and Effects on Survival and Time to Treatment Failure in Patients With Melanoma Treated With Ipilimumab at Memorial Sloan Kettering Cancer Center. J Clin Oncol (2015) 33:3193–8. doi: 10.1200/jco.2015.60.8448 PMC5087335 26282644

[50] Verzoni E Cartenì G Cortesi E Giannarelli D De Giglio A Sabbatini R. Real-World Efficacy and Safety of Nivolumab in Previously-Treated Metastatic Renal Cell Carcinoma, and Association Between Immune-Related Adverse Events and Survival: The Italian Expanded Access Program. J Immunother Cancer (2019) 7:99. doi: 10.1186/s40425-019-0579-z 30944023 PMC6448290

[51] Hussaini S Chehade R Boldt RG Raphael J Blanchette P Maleki Vareki S. Association Between Immune-Related Side Effects and Efficacy and Benefit of Immune Checkpoint Inhibitors - A Systematic Review and Meta-Analysis. Cancer Treat Rev (2021) 92:102134. doi: 10.1016/j.ctrv.2020.102134 33302134

[52] Park R Lopes L Saeed A. Anti-PD-1/L1-Associated Immune-Related Adverse Events as Harbinger of Favorable Clinical Outcome: Systematic Review and Meta-Analysis. Clin Transl Oncol (2021) 23:100–9. doi: 10.1007/s12094-020-02397-5 32495269

[53] Nishino M Giobbie-Hurder A Hatabu H Ramaiya NH Hodi FS. Incidence of Programmed Cell Death 1 Inhibitor-Related Pneumonitis in Patients With Advanced Cancer: A Systematic Review and Meta-Analysis. JAMA Oncol (2016) 2:1607–16. doi: 10.1001/jamaoncol.2016.2453 27540850

[54] Nishino M Ramaiya NH Awad MM Sholl LM Maattala JA Taibi M. PD-1 Inhibitor-Related Pneumonitis in Advanced Cancer Patients: Radiographic Patterns and Clinical Course. Clin Cancer Res (2016) 22:6051–60. doi: 10.1158/1078-0432.ccr-16-1320 PMC5161686 27535979

[55] Naidoo J Wang X Woo KM Iyriboz T Halpenny D Cunningham J. Pneumonitis in Patients Treated With Anti-Programmed Death-1/Programmed Death Ligand 1 Therapy. J Clin Oncol (2017) 35:709–17. doi: 10.1200/jco.2016.68.2005 PMC5559901 27646942

[56] Wang DY Salem JE Cohen JV Chandra S Menzer C Ye F. Fatal Toxic Effects Associated With Immune Checkpoint Inhibitors: A Systematic Review and Meta-Analysis. JAMA Oncol (2018) 4:1721–8. doi: 10.1001/jamaoncol.2018.3923 PMC6440712 30242316

[57] Hobbs KB Yackzan S. Adrenal Insufficiency: Immune Checkpoint Inhibitors and Immune-Related Adverse Event Management. Clin J Oncol Nurs (2020) 24:240–3. doi: 10.1188/20.Cjon.240-243 32441673

[58] Brahmer JR Lacchetti C Schneider BJ Atkins MB Brassil KJ Caterino JM. Management of Immune-Related Adverse Events in Patients Treated With Immune Checkpoint Inhibitor Therapy: American Society of Clinical Oncology Clinical Practice Guideline. J Clin Oncol (2018) 36:1714–68. doi: 10.1200/jco.2017.77.6385 PMC6481621 29442540

[59] Thompson JA Schneider BJ Brahmer J Andrews S Armand P Bhatia S. NCCN Guidelines Insights: Management of Immunotherapy-Related Toxicities, Version 1.2020. J Natl Compr Canc Netw (2020) 18:230–41. doi: 10.6004/jnccn.2020.0012 32135517

[60] Wang D Chen C Gu Y Lu W Zhan P Liu H. Immune-Related Adverse Events Predict the Efficacy of Immune Checkpoint Inhibitors in Lung Cancer Patients: A Meta-Analysis. Front Oncol (2021) 11:631949. doi: 10.3389/fonc.2021.631949 33732650 PMC7958877

