# Supplementary material for: Immune-Related Adverse Events Associated With Outcomes in Patients With NSCLC Treated With Anti-PD-1 Inhibitors: A Systematic Review and Meta-Analysis
Source: Front Oncol. 2021 Sep 15;11:708195. doi: 10.3389/fonc.2021.708195 (PMC8479111; doi:10.3389/fonc.2021.708195)
Supplement: Supplementary file 1 [file DataSheet_1.docx]

Supplementary Material

# Supplementary Figure

**
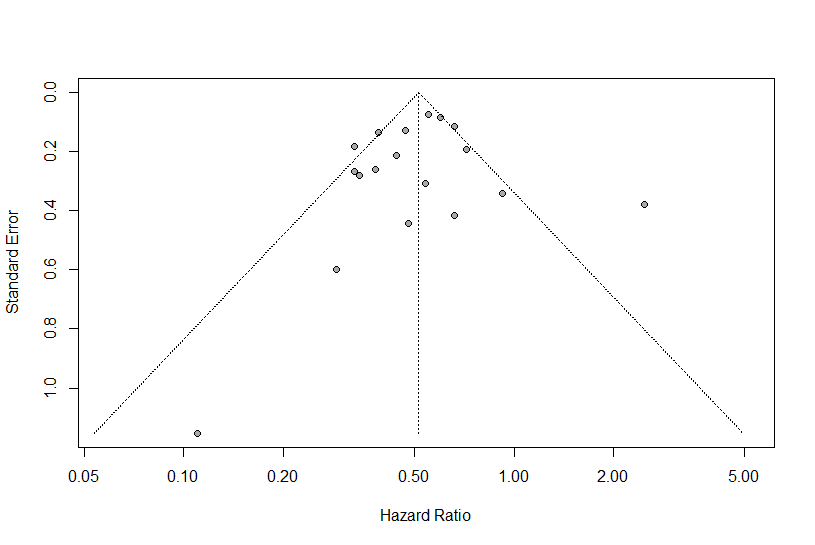
**

**Supplementary Figure S1**. Funnel plot for publication bias of overall survival in patients with NSCLC with and without irAEs treated with anti-PD-1 antibodies.

**
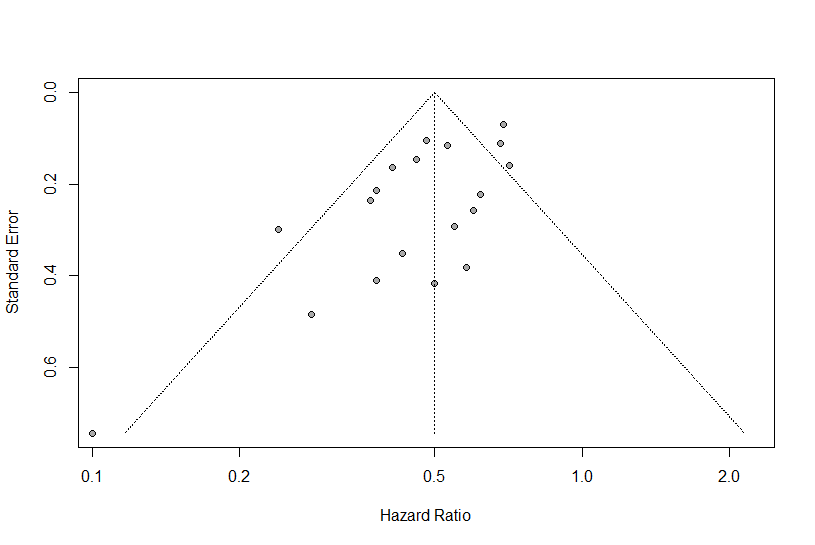
**

**Supplementary Figure S2**. Funnel plot for publication bias of progression-free survival in patients with NSCLC with and without irAEs treated with anti-PD-1 antibodies.

**
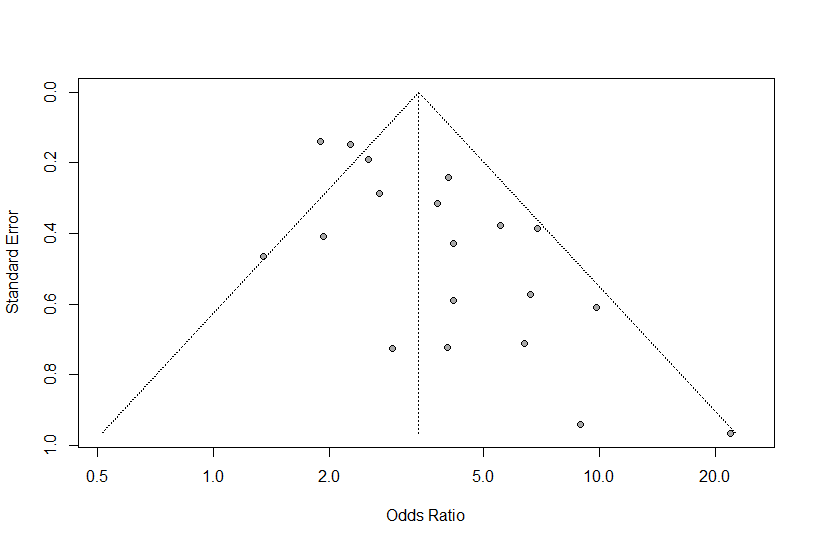
**

**Supplementary Figure S3**. Funnel plot for publication bias of objective response rates in patients with NSCLC with and without irAEs treated with anti-PD-1 antibodies.

**Supplementary Figure S4** Forest plots for sensitivity analysis **
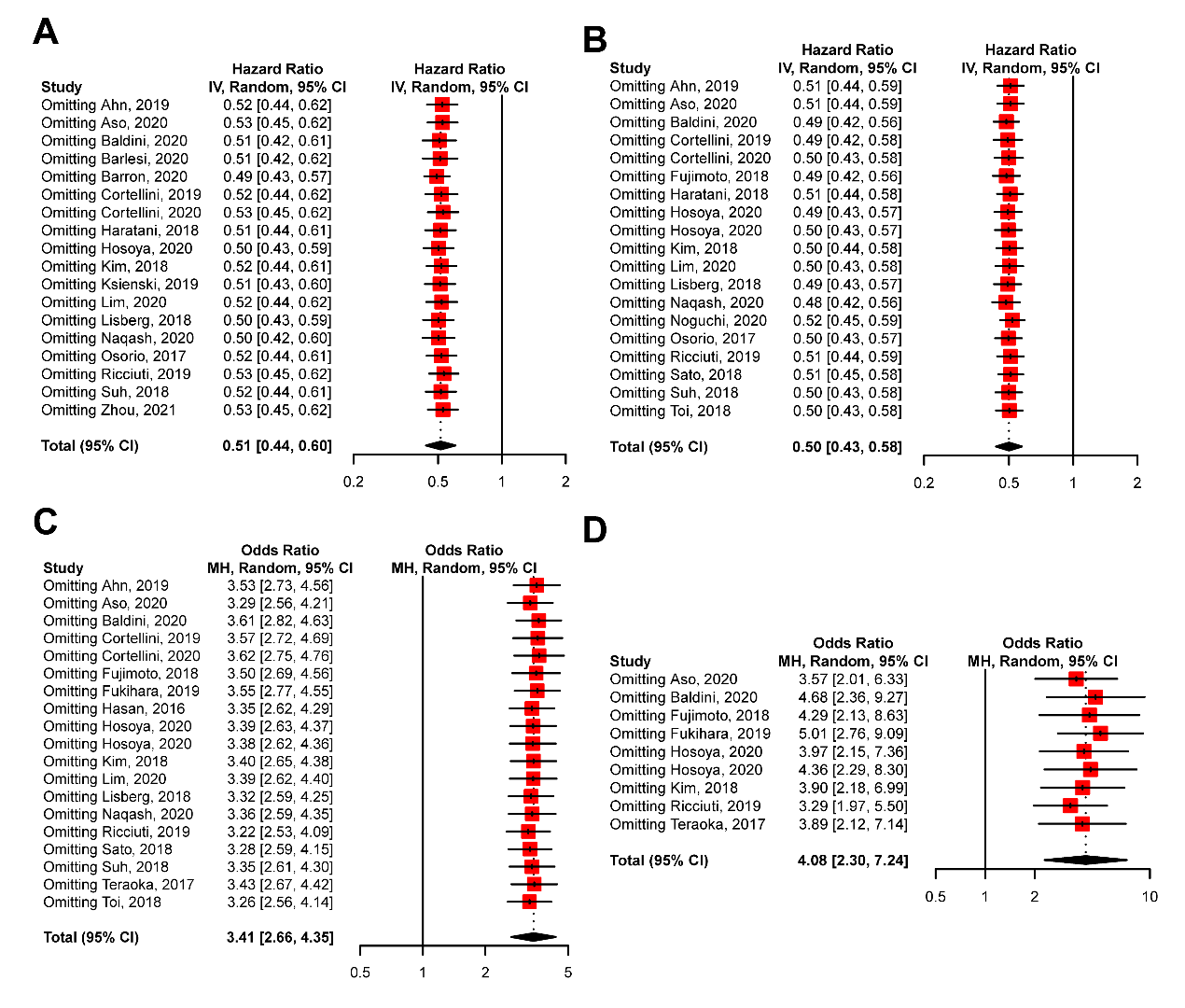
**of overall survival (A), progression-free survival (B), objective response rates (C), and disease control rates (D) in patients with NSCLC with and without irAEs treated with anti-PD-1 antibodies. CI, confidence interval.

**
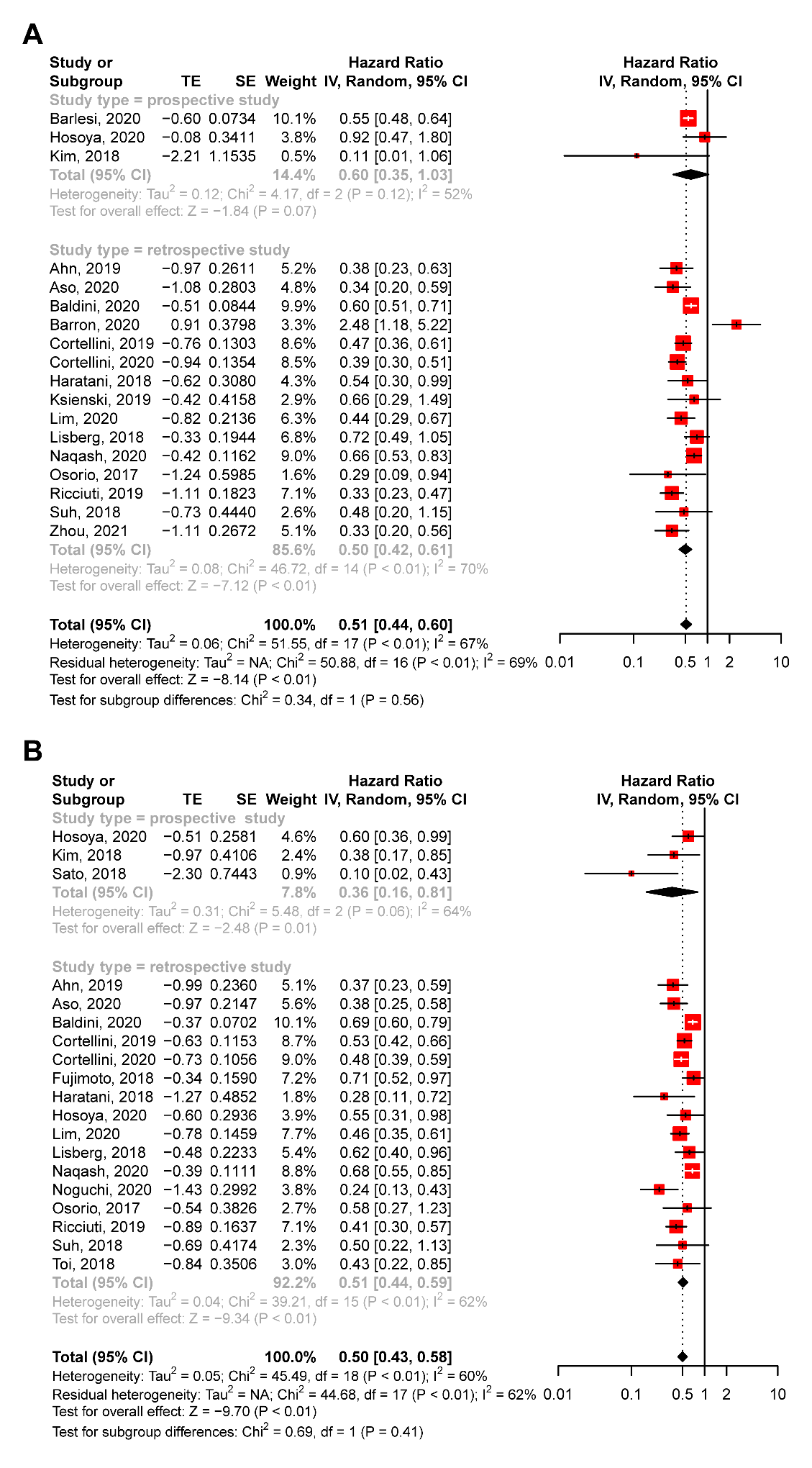
Supplementary Figure S5** Forest plots of subgroup analysis. (A) The association between overall survival and irAEs separated by study types in patients with NSCLC treated with anti-PD-1 antibodies. (B) The association between progression-free survival and irAEs separated by study types in patients with NSCLC receiving anti-PD-1 antibodies. CI, confidence interval.

**Supplementary Figure S6** Forest plots of subgroup analysis. (A) The association between overall survival and irAEs separated by Asian and non-Asian studies in patients with NSCLC treated with anti-PD-1 antibodies. (B) The association between progression-free survival and irAEs separated by Asian and non-Asian studies in patients with NSCLC receiving anti-PD-1 antibodies. CI, confidence interval. **
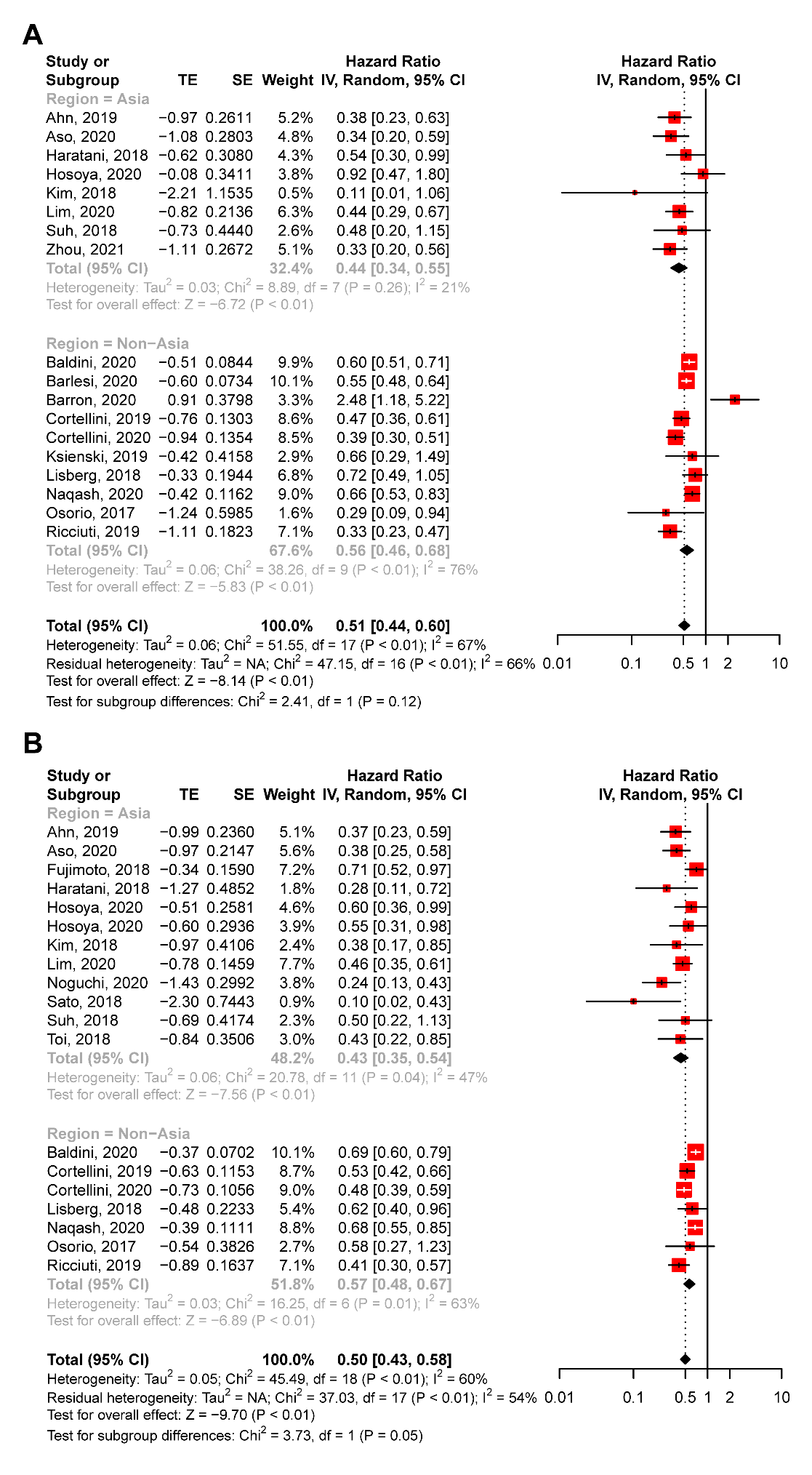
**

**
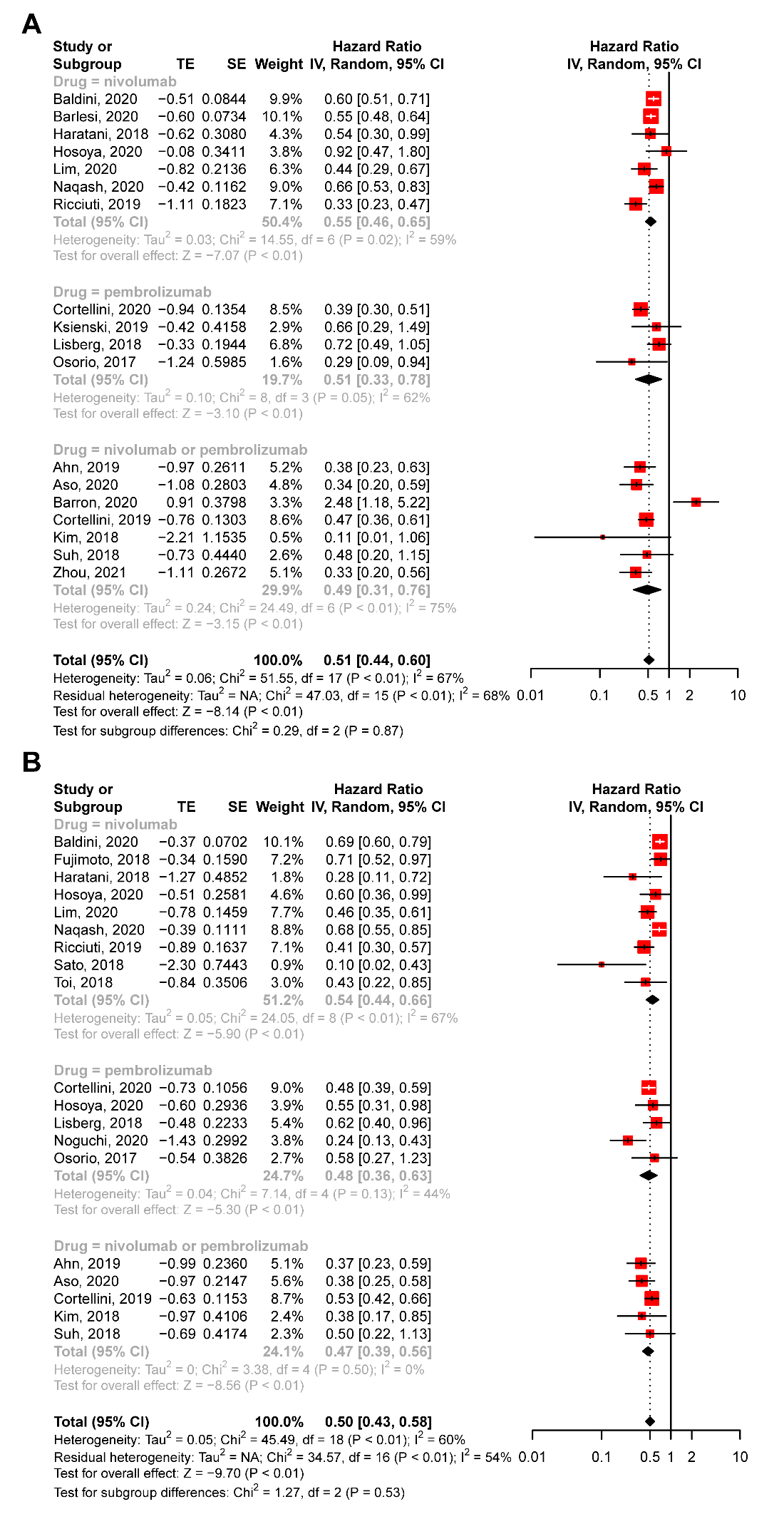
**

**Supplementary Figure S7** Forest plots of subgroup analysis. (A) The association between overall survival and irAEs separated by different drugs in patients with NSCLC treated with anti-PD-1 antibodies. (B) The association between progression-free survival and irAEs separated by different drugs in patients with NSCLC receiving anti-PD-1 antibodies. CI, confidence interval.

# Supplementary Table

**Supplementary Table S1. Search strategies**

| **Set** | **Search** |
| --- | --- |
| #1 | anti-pd1[tw] |
| #2 | anti-pd-1[tw] |
| #3 | "pd1 inhibitor"[tw] |
| #4 | "pd1 inhibitors"[tw] |
| #5 | "pd-1 inhibitor"[tw] |
| #6 | "pd-1 inhibitors"[tw] |
| #7 | pembrolizumab[tw] |
| #8 | keytruda[tw] |
| #9 | nivolumab[tw] |
| #10 | opdivo[tw] |
| #11 | "programmed cell death"[tw] |
| #12 | "immune checkpoint inhibitor"[tw] |
| #13 | "immune checkpoint inhibitors"[tw] |
| #14 | "immune checkpoint blockade"[tw] |
| #15 | "immune checkpoint blockades"[tw] |
| #16 | "Cell Cycle Checkpoints/immunology"[Mesh] |
| #17 | "Programmed Cell Death 1 Receptor/antagonists and inhibitors"[Mesh] |
| #18 | "pembrolizumab" [Supplementary Concept] |
| #19 | "nivolumab" [Supplementary Concept] |
| #20 | #1 OR #2 OR #3 OR #4 OR #5 OR #6 OR #7 OR #8 OR #9 OR #10 OR #11 OR #12 OR #13 OR #14 OR #15 OR #16 OR #17 OR #18 OR #19 |
| #21 | immune related adverse events[tw] |
| #22 | irAEs[tw] |
| #23 | irAE[tw] |
| #24 | toxicity[tw] |
| #25 | safety[tw] |
| #26 | adverse event[tw] |
| #27 | adverse effect[tw] |
| #28 | side effect[tw] |
| #29 | #21 OR #22 OR #23 OR #24 OR #25 OR #26 OR #27 OR #28 |
| #30 | outcome[tw] |
| #31 | efficacy[tw] |
| #32 | association[tw] |
| #33 | correlation[tw] |
| #34 | response[tw] |
| #35 | prognosis[tw] |
| #36 | survival[tw] |
| #37 | OS[tw] |
| #38 | PFS[tw] |
| #39 | ORR[tw] |
| #40 | #30 OR #31 OR #32 OR #33 OR #34 OR #35 OR #36 OR #37 OR #38 OR #39 |
| #41 | non small cell lung cancer[tw] |
| #42 | NSCLC[tw] |
| #43 | “carcinoma, non-small-cell lung”[Mesh] |
| #44 | #41 OR #42 OR #43 |
| #45 | #20 AND #29 AND #40 AND #44 |

**Supplementary Table S2.** Characteristics of included studies

| Author/year | Clinical stage | Histological type | PD-L1 expression | Driver gene mutation |
| --- | --- | --- | --- | --- |
| Ahn/2019 | Advanced | SCC 47 (30.3); ADC 105 (67.7); Other 3 (1.9) | ≥50%: 53 (34.2); 1-49%: 46 (29.7); <1%: 32 (20.6); Unknown: 24 (15.5) | EGFR or ALK: 23 (14.8) |
| Aso/2020 | Advanced | SCC 55 (35.5); Non-SCC 100 (64.5) | >50%: 33 (21.3); 1-50%: 35 (22.6); <1%: 22 (14.2); Unknown: 65 (41.9) | EGFR: 17 (11.0) |
| Baldini/2020 | Stage IIIB/IV | SCC 371 (18.9); Non-SCC 1588 (81.1) | / | EGFR: 101 (6.3); EML4-ALK: 14 (0.8) |
| Barlesi/2020 | Stage III/IV | SCC 437 (30.8); Non-SCC 983 (69.2) | / | / |
| Barron/2020 | Stage III/IV | SCC 11 (10.9); ADC 85 (84.1); Other 5 (5.0) | Positive ^a^: 27 (26.7); Negative ^a^: 9 (8.9); Unknown: 65 (64.4) | EGFR: 16 (15.8); ALK: 0; Kras: 0 |
| Cortellini/2019 | Advanced | SCC 235 (42.0); Non-SCC 324 (58.0) | ≥50%: 100 (17.9); 1-49%: 60 (10.7); <1%: 45 (8.1); Unknown: 354 (63.3) | / |
| Cortellini/2020 | Stage IV | SCC 246 (24.4); Non-SCC 764 (75.6) | ≥50%: 1010 (100) | / |
| Fujimoto/2018 | Stage IIIB/IV | SCC 135 (22.0); ADC 413 (67.4); Other 65 (10.6) | / | EGFR: 95 (15.5); ALK: 12 (2.0) |
| Fukihara/2019 | Recurrent or Advanced | SCC 144 (84.7); Non-SCC 26 (15.3) | / | / |
| Haratani/2018 | Recurrent or Advanced (IIIB/IV) | SCC 33 (24.6); Non-SCC 101 (75.4) | / | EGFR: 30 (22.4); ALK: 6 (4.5) |
| Hasan/2016 | Advanced | SCC 14 (34.1); ADC 23 (56.1); Other 3 (7.3) | / | / |
| Hosoya/2020 | Recurrent or Advanced (IIIB/IV) | SCC 34 (23.0); Non-SCC 114 (77.0) | ≥50%: 148 (100) | EGFR: 5 (3.4); ALK: 0 |
| Hosoya/2020 | Recurrent or Advanced (IIIB/IV) | SCC 16 (21.1); Non-SCC 60 (78.9) | ≥75%: 8 (10.5); 50-74%: 9 (11.8); <50%: 44 (57.9); Unknown: 15 (19.7) | EGFR: 12 (15.8); ALK: 2 (2.6) |
| Kim/2018 | Stage IV | SCC 20 (34.5); Non-SCC 38 (65.5) | / | / |
| Ksienski/2019 | Recurrent or Advanced (IIIB/IV) | SCC 42 (22.1); Non-SCC 148 (77.9) | ≥50%: 176 (92.6); 1-49%: 14 (7.4) | EGFR: 7(3.7); ALK: 0 |
| Lim/2020 | Stage III/IV | SCC 85 (28.4); ADC 198 (66.2); Other 16 (5.4) | / | EGFR: 48 (16.1); ALK: 5 (1.7) |
| Lisberg/2018 | Stage III/IV | SCC 19 (19.6); Non-SCC 78 (80.4) | ≥50%: 17 (17.5); 1-49%: 38 (39.2); <1%: 21 (21.6); Unknown: 21 (21.6) | EGFR: 30 (30.9); ALK: 2 (2.1) |
| Naqash/2020 | Stage IV | SCC 146 (27.5); Non-SCC 385 (72.5) | ≥50%: 25 (4.7); 1-49%: 44 (8.3); <1%: 95 (17.9); Unknown: 367 (69.1) | EGFR: 34 (6.4); ALK: 11 (2.1); KRAS: 87 (16.4); TP53: 45 (8.5) |
| Noguchi/2020 | / | SCC 23 (24.5); Non-SCC 71 (75.5) | ≥50%: 71 (75.5); 1-49%: 23 (24.5) | / |
| Osorio/2017 | Stage IV | / | / | / |
| Ricciuti/2019 | Recurrent or Advanced | SCC 41 (21.0); Non-SCC 154 (79.0) | ≥1%: 13 (6.7); <1%: 38 (19.5); Unknown: 144 (73.8) | EGFR: 16 (8.2); ALK: 1 (0.5) |
| Sato/2018 | Recurrent or Advanced (IIIB/IV) | SCC 10 (26.3); Non-SCC 28 (73.7) | ≥50%: 13 (34.2); 1-49%: 20 (52.6); <1%: 5 (13.2) | EGFR: 6 (15.8); ALK: 1 (2.6) |
| Suh/2018 | Recurrent or Advanced (IIIB/IV) | SCC 17 (31.5); Non-SCC 37 (68.5) | ≥50%: 7 (13.0); <50%: 29 (53.7); Unknown: 18 (33.3) | / |
| Teraoka/2017 | Stage IIIB/IV | SCC 9 (20.9); ADC 30 (69.8); Other 4 (9.3) | ≥50%: 8 (18.6); 1-49%: 10 (23.3); <1%: 17 (39.5); Unknown: 8 (18.6) | EGFR: 7 (16.3); ALK: 1 (2.3) |
| Toi/2018 | Advanced | SCC 27 (38.6); Non-SCC 43 (61.4) | / | EGFR: 5 (7.1) |
| Zhou/2021 | Recurrent or Advanced (IIIB/IV) | SCC 69 (36.1); ADC 105 (55.0); Other 17 (8.9) | ≥50%: 52 (27.2); 1-49%: 34 (17.8); <1%: 50 (26.2); Unknown: 71 (37.2) | / |

1. The cut-off value for positive PD-L1 expression was not stated in the article.

For histological type, PD-L1 expression, and driver gene mutation, data were illustrated as patient number (%). ADC, adenocarcinoma; ALK, anaplastic lymphoma kinase; EGFR, Epidermal Growth Factor Receptor; non-SCC, non-squamous cell carcinoma; PD-L1, programmed death ligand 1; SCC, squamous cell carcinoma.
